# Non-Pharmacological Pain Management in Labor: A Systematic Review

**DOI:** 10.3390/jcm12237203

**Published:** 2023-11-21

**Authors:** Wassan Nori, Mustafa Ali Kassim Kassim, Zeena Raad Helmi, Alexandru Cosmin Pantazi, Dragos Brezeanu, Ana Maria Brezeanu, Roxana Cleopatra Penciu, Lucian Serbanescu

**Affiliations:** 1 College of Medicine, Mustansiriyah University, Baghdad 10052, Iraq; zeena.helmi@uomustansiriyah.edu.iq; 2 Faculty of Medicine, “Ovidius” University of Constanta, 900470 Constanta, Romania; brezeanudragos@gmail.com (D.B.); anmariataras@gmail.com (A.M.B.); roxanapenciu@yahoo.com (R.C.P.); lucian_trocadero@yahoo.com (L.S.); 3 Obstetrics and Gynecology Department, Clinical Emergency Hospital of Constanta, 900591 Constanta, Romania

**Keywords:** non-pharmacological pain management, pregnancy, mothers, labor, obstetrician efficacy, side effects

## Abstract

Childbirth is a remarkable, life-changing process and is frequently regarded as an excruciating, physically and emotionally demanding experience that women endure. Labor pain management poses a significant challenge for obstetricians and expectant mothers. Although pharmacological pain management is the gold standard, it still imposes risks on the mother and baby. Recently, non-pharmacological pain management (NPPM) has emerged as a safe, effective option. Six databases were searched for articles published up to 2023 using specific related keywords and defined inclusion and exclusion criteria. The extraction and gathering of data was made so as to be categorized into physical, psychological, and complementary NPPM techniques. In light of the enormous development and diversity of NPPM techniques, the present review aims to examine contemporary NPPM knowledge and application, discussing efficacy, advantages, limitations, and potential adverse effects, with a specific focus on women’s individual requirements, to strengthen obstetricians’ knowledge in guiding decision-making for women in childbirth.

## 1. Introduction

Historically, labor pain has been recognized as an inherent part of childbirth, although approaches to its management have varied across cultures and time periods [1]. With the advent of modern medicine, the focus shifted toward pharmacological interventions. By the late 19th century, interventions such as chloroform and ether were used for labor pain, followed by the introduction of “twilight sleep” in the early 20th century—a combination of morphine and scopolamine that induced a state of semi-consciousness [2,3].

In the latter half of the 20th century, advances in anesthesia led to the widespread use of regional analgesics, such as epidurals and spinal blocks, for labor pain [4]. These methods became the gold standard in many high-income countries due to their effectiveness in reducing pain [5]. In recent decades, the use of opioids such as fentanyl and morphine has also become common [6]. Although they do not eliminate pain, they can help to make it more manageable. However, pharmacological pain management methods (PPM) have been associated with various side effects and risks despite their effectiveness. For example, epidurals can lead to a drop in blood pressure, fever, and a raised need for assisted delivery [7]. Moreover, they lengthen the labor’s duration. Opioids induce nausea and affect the neonate (breathing and heart tracing) if admitted too soon [8,9]. Pain is the norm of childbirth; reducing pain via drugs makes laboring women lose essential feedback, potentially leading to more prolonged labor or increased intervention. Some forms of PPM reduce a woman’s motility or the ability to take different positions to alleviate discomfort; this lack of control over pain is distressing to women [10]. Over the past few decades, a growing interest has been expressed in revisiting non-pharmacological pain management techniques (NPPM) to reduce labor pain [11,12]. This shift is driven by a confluence of factors, including increasing evidence of pharmacological interventions’ side effects and risks. Additionally, there has been a broader societal shift towards more patient-centered and holistic healthcare, emphasizing personal autonomy, shared decision-making, and natural and complementary therapies [13]. These trends have led to an increased interest in NPPM, which relieves pain and empowers women to actively engage in the birth experience. NPPMs have demonstrated encouraging outcomes in diminishing pain intensity and enhancing satisfaction and are commonly regarded as safe, with minimal adverse effects compared to pharmacological interventions [3,5,8]. In light of the vast improvement and diversity of NPPM techniques, the current review aimed to investigate up-to-date NPPM understanding and application, specifically focusing on women’s individual needs, regarding NPPM’s effectiveness, advantages, limitations, and potential adverse effects, to provide obstetricians with the necessary knowledge to guide advice and decision-making for women during childbirth.

## 2. Understanding Pain in Labor

The nature of pain experienced during labor undergoes modifications as the process progresses. During the first stage of labor, the primary source of pain is visceral in nature, originating mostly from the cervix, uterus, and adnexa. This pain is mediated by sympathetic fibers that transmit signals to the ganglia of the posterior nerve roots located at the T10-L1 spinal levels [5]. During the late first stage and early second stage of labor, pain arises from the distention and traction of the pelvic organs. The pudendal nerve is responsible for transmitting pain signals to the ganglia of the posterior nerve roots located at spinal levels S2 to S4. During the second stage of labor, the sensation of pain is elicited by the stretching of the perineal structures as the fetus descends [5,11]. Comprehending the complexities of labor pain goes beyond the physiological aspects; it necessitates an understanding of psychological and socio-cultural elements. It is crucial to grasp the multifaceted nature of labor pain to assess NPPM better. From a physiological standpoint, uterine contractions and cervical dilation are the main causes of labor pain, because they activate pain receptors (nociceptors) and send signals to the brain [11]. The intensity of labor pain can vary greatly among women, between different labors in the same woman, and it is affected by various factors such as the baby’s position, size, and the speed of labor [12]. On a psychological level, labor pain is influenced by a woman’s emotions, expectations, and previous experiences [13,14]. Fear and anxiety can heighten pain perception by increasing tension and resistance. As confidence, relaxation, the feeling of control in their labor, and continuous support are all less likely to result in severe pain, the women are more likely to cope and have a positive birth experience [15,16,17,18]. Psychological preparation for childbirth can reduce the need for analgesia and increase satisfaction with pain management [19,20]. Socio-cultural factors, cultural beliefs, and societal attitudes toward childbirth can influence a woman’s expectations and coping strategies. In some cultures, labor pain is viewed as a natural and empowering part of childbirth, while in others, it is seen as something to be avoided or feared [21,22]. Furthermore, social support is crucial during labor. Having a supportive companion can significantly improve a woman’s experience of pain and reduce her need for pharmacological analgesia [23,24].

## 3. Materials and Methods

**Figure 1:**
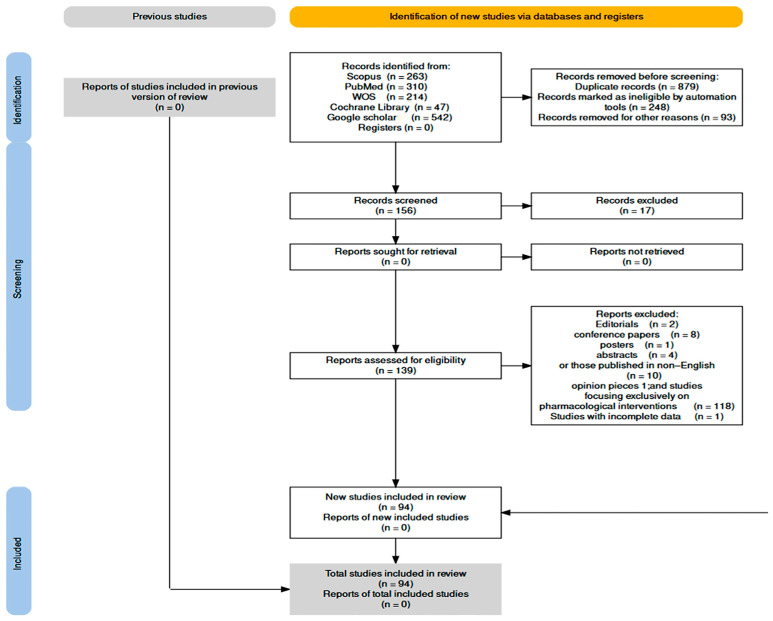
A PRISMA flow diagram for searching and selecting eligible studies included in the review.

The research protocol was developed in accordance with the PRISMA criteria [25]. We searched online through five digital repositories: PubMed, Scopus, Web of Science, Google Scholar, and the Cochrane Library. The search technique included a hybrid approach, incorporating keywords and subject headings about NPPM, pain management, and women in childbirth. The search results were refined by combining the keywords (non-pharmacological technique, pregnancy, mothers, labor, pain management, obstetrician) with Boolean operators such as “and” and “or.” We searched peer-reviewed literature published till 20 July 2023. The study methodology is demonstrated in the study flow chart [26]; see Figure 1.

The results obtained were examined by scanning and screened by title and abstract following PICO (population, intervention, control, and outcome) criteria [27].

The population of interest was primarily pregnant >18 years old during childbirth. We focused on interventions that reduced labor pain and shortened labor duration. The NPPM interventions were further stratified based on their action mode into physical, psychological, and complementary techniques. For each intervention, a comparison was made for the underlying action mechanism and benefits. A discussion was conducted concerning their efficiency, benefits, challenges, and limitations. Study selection was conducted by two reviewers who worked independently. This selection process adhered to specific criteria for inclusion and exclusion.

An exclusion criterion was made to remove duplicate results and research that did not meet the eligibility criteria. Our study included published research specifically centered on NPPM for managing labor pain. Additional exclusions were made for case reports, editorials, opinion pieces, and studies solely concentrating on pharmaceutical therapies.

A total of (n = 94) papers met the eligibility criteria and were then subjected to analysis. The studies were categorized according to the specific NPPM investigated into physical (n = 38), psychological (n = 29), and complementary treatments (n = 27). The data extraction from the chosen studies encompassed identifying and recording the authors’ names, the year of publication, the study’s design, the size of the sample, a description of the intervention, the main findings, and the conclusions drawn. Each intervention’s effectiveness, advantages, and limitations were evaluated and integrated using a systematic synthesis technique.

## 4. Categorization of Non-Pharmacological Methods for Pain Relief in Labor

These can be categorized based on the mechanisms of action into physical, psychological, and complementary techniques.

### 4.1. Physical Modalities

There are several physical methods listed under NPPM during labor. These methods include massage, pressure on precise anatomical locations, Transcutaneous Electrical Nerve Stimulation (TENS), water immersion, heat and cold therapy, breathing techniques, positioning, and movement [28,29]. The sub-types of each method, mechanism of action, perceived benefits, and supporting references are all summarized in Table 1.

**Table 1:** Non-pharmacological pain management in labor: An in-depth analysis of physical modalities concerning the mechanism of action, perceived benefit, and the supporting references.

Methods	Methods Sub-Types	Proposed Mechanism of Action	Perceived Benefit	Authors’ Name; Publication Year
Massage	▪ Vibrating ▪ Stroking ▪ Effleurage	▪ Gentle massage or counter-pressure to specific areas is effective in reducing discomfort and triggering endorphin releases, an endogenous hormone with analgesic properties. ▪ Additionally, it promotes a subjective sense of psychological relief.	▪ It proved effective in reducing lab pain, yet the character of pain and lab duration was unchanged. ▪ Combining oil with massage decreased lab pain and duration and improved satisfaction.	Pawale et al. [30]; 2020 Silva Gallo et al. [31]: 2013 Eskandari F et al. [32]; 2022
Pressure on precise anatomical locations	▪ Acupressure ▪ Acupuncture: ▪ traditional acupuncture ▪ sham acupuncture	▪ The application of pressure on precise anatomical locations potentially induces Relaxation and reduces Stress. ▪ Triggering non-painful stimuli closes spinal cord “gates,” thus blocking pain signals. ▪ Triggering acupuncture spots sends signals to the brain to release endorphins. ▪ Regulation of oxytocin hormones ▪ For more details, see Figure 2.	▪ Acupressure may improve women’s satisfaction and reduce labor pain and duration. ▪ There is insufficient evidence of Acupuncture’s effects on labor; it seems that it decreases pain intensity but not duration and has better satisfaction rates.	Smith et al. [33]; 2020 Schlaeger et al. [34]; 2017 Eshraghi et al. [35]; 2021
Transcutaneous Electrical Nerve Stimulation (TENS)	▪ Conventional TENS: reduces labor pain. ▪ Acupuncture TENS ▪ Intense TENS ▪ Burst mode TENS ▪ Modulated TENS	▪ Applying low-intensity electrical pulses to targeted regions of the body via electrodes affixed to the skin. ▪ It inhibits the pain signals’ transmission to the nervous system.	▪ It significantly reduces pain intensity; however, the evidence was low. ▪ A significant reduction in pain score and improved women’s satisfaction.	Thuvarakan et al. [36]; 2020 Gibson et al. [37]; 2019 Daniel et al. [38]; 2021
Water immersion	▪ Cold water ▪ Hot water ▪ Alternating hot/cold water bathing	▪ Immersing in a bath or utilizing a birthing pool can induce relaxation, diminish pain perception, and facilitate smoother movement during childbirth.	▪ There was low evidence that immersion reduces the need for PPM. ▪ Significant improvement in physical and psychological comfort, and the need for pain relief.	Cluett et al. [39]; 2018 Carlsson et al. [40]; 2020 Cooper et al. [41]; 2022
Heat therapy	▪ Warm pack and towels ▪ Hot water bags ▪ Warm shower ▪ Thermal and infra-red belt	▪ Administration of heat to a specific region experiencing pain enhances blood circulation, induces muscle relaxation, and alleviates pain perception.	▪ Evidence confirmed an effective reduction in labor pain intensity and labor duration. ▪ Significant reduction in post-labor pain	Goswami et al. [42]; 2022 Akbarzadeh et al. [43]; 2018 Akbarzadeh et al. [44]; 2016 Dastjerd et al. [45]; 2023
Cold therapy	▪ Ice packs ▪ Ice massage	Utilization of cold packs or ice to induce numbness in a specific area, thereby mitigating inflammation and offering temporary pain alleviation.	▪ Significant reduction in pain intensity and duration.	Shirvani et al. [46]; 2014 Emine et al. [47]; 2022 Serap et al. [48]; 2022
Breathing techniques	▪ Deep, slow, and patterned breathing.	▪ Effective in diverting attention from pain and facilitating a state of relaxation.	▪ Effective reduction in labor pain added to a shorter labor duration. ▪ Ineffective in reducing pain in the 1st stage.	Baljon et al. [49]; 2022 Issac et al. [50]; 2023 Yuksel H et al. [51]; 2017 Boaviagem et al. [52]; 2017
Positioning and Movement	▪ Changing positions frequently, such as walking, squatting position, ▪ Birthing ball rocking	▪ Helps manage pain by utilizing gravity and promoting optimal fetal positioning.	▪ Effective in reducing pain and duration of labor. ▪ Upright positions and free mobility reduce labor duration and pain and improve women’s satisfaction.	Huang et al. [53]; 2019 Ondeck et al. [54]; 2019 Borges et al. [55]; 2021 Ali SA et al. [56]; 2018

### 4.2. Psychological Techniques

Cognitive Behavioral Therapy (CBT) aims to identify and modify maladaptive thoughts, emotions, and behaviors. Moreover, CBT assists individuals in cultivating a perception of control in managing pain, fostering the acquisition of pain-coping strategies, and enhancing self-esteem [57]. CBT was used to manage labor pain; there was inconsistency in the reported literature; some discussed reduced psychological aspects of pain and improved satisfaction [58]. However, pain medication was still needed.

Others have discussed how CBT techniques significantly reduced pain intensity and labor duration [57].

Cognitive behavioral therapy helps individuals have a sense of control in coping with pain, develop pain-coping behaviors, and increase self-respect [57,59]. The main methods of CBT include:

- Relaxation techniques;
- Virtual reality (VR);
- Music;
- Distraction technique.

The main mechanism and benefits of each are summarized in Table 2.

**Table 2:** Summary of non-pharmacological pain management in labor: An in-depth analysis of psychological modalities, concerning the mechanism of action, perceived benefit, and the supporting references.

Methods	Methods Sub-Types	Proposed Mechanism of Action	Perceived Benefit	Authors’ Name; Publication Year
Relaxation technique	Relaxation, Yoga, and Music Mindfulness and audio-analgesia	Progressive muscle relaxation, guided imagery, and visualization have been found to be effective in mitigating anxiety and fostering tranquility throughout labor.	Reduction in pain intensity. Empowers women with sense of control. Improves satisfaction with the birth experience	Smith et al. [15]; 2018 Zhang et al. [60] Jahdi et al. [61]; 2017
Virtual reality (VR)	Interactive VR game VR meditation VR-guided meditation VR mindfulness	Modulating pain perception by interfering with psychological factors Distraction which reduces the perception of pain	Reduces pain intensity and anxiety during childbirth. Enhanced satisfaction with the birth experience. No effect on labor duration.	Massov et al. [62]; 2021 Musters et al. [63]; 2023 Baradwan et al. [64]; 2022Xu et al. [65]; 2022
Music	--	Modulation of pain responses and neuronal activity in CNS while engaging with music. Increases pain tolerance and lowers pain intensity. For further details, see Figure 3.	They experience natural delivery with less stress and less medication. Reduces pain score by 3.4 times.	Timmerman et al. [66]; 2023 Estrella-Juarez et al. [67]; 2023 Chehreh et al. [68]; 2023 García González et al. [69]; 2018
Distraction	Counting numbers Remembering poetry, a pleasant memory, or a joke Using vulgar cards	Minds occupied by excitement are distracted from the excitement of the pain senses. he technique reduces the pain effect on the CNS and nerve transmitters	Reduction in labor pain and stress No reduction in labor duration	Ireland et al. [70]; 2016 Amiri et al. [71]; 2019 Melillo et al. [72]; 2022

### 4.3. Complementary and Alternative Approaches

Over the past decade, there has been a growing scholarly focus on literature examining the role of Complementary and Alternative Approaches (CAA) in mitigating pain during childbirth [73]. CAA exhibits a higher prevalence among women within the reproductive age range [74]. The utilization of this intervention during childbirth is quite prevalent, as indicated by a survey conducted in Australia, with a reported rate of 75% [75]. Complementary and Alternative Medicine is a term employed by the U.S. National Center for Complementary and Integrative Health to denote a range of practices that can be utilized in conjunction with conventional and established medical care (complementary) or as a substitute for it (alternative) [76]. An in-depth analysis of complementary and alternative approaches concerning the mechanism of action, perceived benefit, and the supporting references [77,78,79,80,81,82,83,84,85,86,87,88,89,90,91,92] are summarized in Table 3.

## 5. Discussion

This section will discuss what is essential for obstetricians to know to guide the maternal decision in choosing NPPM. The efficiency and benefits of NPPM will be addressed first, as well as the associated challenges in practice. Then a discussion will be conducted on the reported side effects. Finally, we will suggest future research in the field.

### 5.1. The Efficiency of Non-Pharmacological Pain Management Techniques in Reducing Labor Pain

Non-pharmacological pain management approaches offer a multitude of benefits to laboring women. The choice of pain management methods should be patient-centered. Every woman has unique needs and preferences, and these should guide the decision-making process. However, NPPM’s effectiveness differs among women due to individual preferences, educational status, parity, culture, and clinical settings [93,94]. Smith et al. [95] explored the experiences of women who utilized NPPM during labor. Women reported a wide range of experiences, ranging from empowerment and satisfaction to feelings of failure when the techniques were ineffective, which highlights the importance of tailored interventions that consider individual needs and preferences and emphasize realistic expectations [95].

Many studies have declared that PPM techniques may be more effective than NPPM in reducing pain intensity [96,97,98]. Even the maternal evaluation of NPPM revealed less efficacy than expected [99]. Epidural anesthesia and other types of neuraxial analgesia are still considered the “gold standard” for alleviating labor pain [100,101]. Beyable et al.’s systemic review set a pain score of less than 3 to obtain adequate pain relief via NPPM and advised PPM for a score of more than 3. The authors combined NPPM with a lower dose of standard pain-relieving medications to improve maternal pain relief while minimizing neonatal adverse effects [98].

A growing body of research has demonstrated that continuous labor support substantially reduces medical interventions, the need for PPM, and operative delivery [18]. Engaging in discussions with healthcare professionals or participating in anti-natal labor education classes is advisable to develop effective coping strategies and acquire the necessary knowledge and proficiency in implementing these techniques [94,102]. Earlier work in the field has addressed the efficacy of NPPM compared to controls and/or placebo in RCTs, showing variable degrees of benefits [30,33,35,38,40,42,43,51]. Others combined more than one method of NPPM and showed higher success rates [32,45,48,49,56,67].

Hu et al.’s [95] meta-analysis evaluated the efficacy of various NPPM techniques; their results indicated that acupressure, aromatherapy, and massage had the highest efficacy in reducing lab pain compared to standard care. While compared to placebo, only acupressure and aroma were statistically significant [95]. Rank probability tests for reducing labor pain intensity confirmed that aromatherapy, acupressure, and TENS ranked the highest with probabilities of 35, 31, and 15%, respectively [96]. The results obtained were in good agreement with earlier studies [103,104,105].

**Figure 4:**
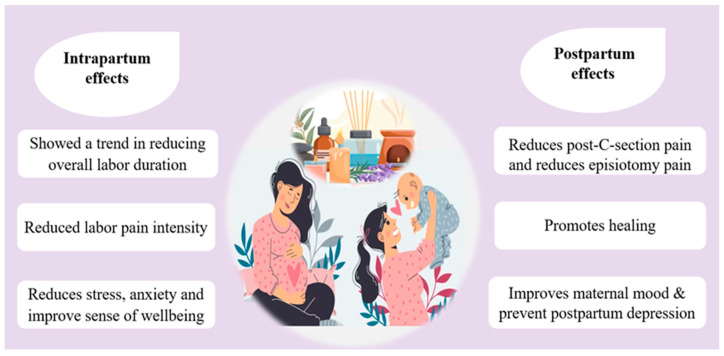
Shows the beneficial effect of aromatherapy during and after childbirth.

According to Liao et al. [104], aroma’s beneficial effects extend beyond labor pain, as summarized in Figure 4.

Chen et al. [105] declared that aroma effectively reduced labor pain intensity and showed a trend toward reducing overall labor duration. Yoga ranked first in reducing first stage labor duration. While acupressure, massage, and yoga reduced the duration of the second stage of labor, respectively, based on Hu et al.’s results [96], Melillo et al.’s [72] systemic review examined the strengths of many NPPM methods in reducing labor pain during the first stage and compared their effects. Their results confirm that massage, birth ball, acupressure, and distraction had a large and significant effect (*p* = 0.001) on reducing labor pain intensity. At the same time, heat was moderately effective. The author pointed out that although many of these methods effectively minimize the intensity of labor pain, standardization of the techniques necessitates additional high-quality RCTs [72].

### 5.2. Benefits of Non-Pharmacological Pain Management

The mounting evidence on the benefits of NPPM approaches in labor pain holds significant implications and benefits in practice. Understanding these benefits can guide healthcare providers in helping women make informed decisions about their labor [99].

NPPM offers a better safety profile than PPM. One of the main advantages of non-pharmacological techniques is the reduction in potential side effects associated with pharmacological interventions [106]. Strategies like incorporating movement and maintaining an upright posture are known to promote favorable fetal positioning and enhance the effectiveness of contractions, thereby contributing to an easier, shorter labor time and an expeditious labor experience [53,107]. NPPM techniques such as “breathing exercises, massage, water immersion, and relaxation techniques provide a calm and relaxed setting”, thus alleviating stress and its related consequences in laboring women and the unborn baby. Moreover, they tend to lessen urgent and operative interventions and make women favor a natural birth experience [108,109].

Water immersion significantly reduced intrapartum blood loss; the effect was mediated by improving circulation and maintaining CVS stability [40,110]. Physical techniques such as massage, changing positions, and controlled pushing have been shown to reduce the risk of vaginal tears. This contributes to rapid and smooth post-partum recovery [111]. NPPM increases the sense of control through active participation by the laboring woman. Whether women are using breathing techniques, assuming comfortable positions, or utilizing visualization, these methods provide a sense of control over the labor process [99].

Enhanced satisfaction with the birth experience is another advantage; research shows that women who use non-pharmacological pain management techniques report higher satisfaction with their childbirth experience [112]. This may be due to a combination of feeling in control, being actively involved in decision-making, and perceiving labor as a natural process [74].

NPPM interventions are of paramount importance in the prevention and amelioration of post-partum depression. By facilitating relaxation and enhancing emotional well-being throughout the process of childbirth, these interventions have the potential to exert a positive influence on a woman’s mental health in the post-partum period. Music therapy has the potential to facilitate a positive childbirth experience, thereby potentially reducing the incidence of depression [61,69,113].

### 5.3. Challenges of Non-Pharmacological Pain Management

During labor, NPPMs are frequently used to assist women in coping with childbirth agony and distress. Although these techniques are potentially effective, there are several obstacles and challenges that limit their use in practice [99]. Insufficient knowledge and understanding among healthcare professionals, inadequate training, and limited resources collectively hinder NPPM’s incorporation into obstetric care. Moreover, the prevailing tendency to prioritize pharmacological interventions further contributes to the inadequate implementation of NPPM. Additionally, some healthcare contexts lack these resources, limiting women’s access to them [106]. Many women may not have sufficient knowledge and education regarding NPPM. For that reason, they might feel insecure in using them or not comprehend their advantages [114,115]. Cultural and societal expectations surrounding childbirth can impact a woman’s choice to use NPPM. Some cultures may consider the use of medicine during labor essential, making it difficult for women to opt for alternative methods without facing criticism [22]. This highlights the necessity for a fundamental change in healthcare paradigms, advocating for the prioritization and thorough integration of NPPM in order to enhance the experience of giving birth for a mother.

Each woman’s labor experience is unique; what is successful for one woman could prove unsuccessful for another. Due to personal preference or previous bad experiences with NPPM, some women might favor pharmacological pain relief methods [99]. Support and encouragement from healthcare personnel are crucial to a woman’s ability to use NPPM successfully. If medical professionals are unfamiliar with these methods or fail to encourage their use, women may feel unsupported in their decision [116].

It is essential to acknowledge that labor pain can be excruciating and overwhelming for some women, making it difficult to depend solely on NPPM for pain relief. That especially applies to cases of protracted or intense labor; women might need additional pharmacological pain options to endure pain [117]. Finally, labor is an emotionally and psychologically taxing process. Women in labor may find it difficult to concentrate on NPPM if they are anxious or exhausted [118].

To sum up, non-pharmacological pain interventions present numerous advantages within the realm of labor-related complications. Effective implementation in practical settings necessitates the development of a comprehensive approach, including enhancing women’s education and support, improving the accessibility of resources, and promoting the dissemination of knowledge regarding NPPM among healthcare providers.

### 5.4. Side Effects of Non-Pharmacological Methods

There is general awareness regarding the safety of NPPM techniques. Few side effects were reported in practice following their use. It is important to remember that not every woman experiences the side effects listed. Before incorporating NPPM, these side effects must be discussed with expecting mothers [96,105]. Some mothers may not obtain enough pain relief from the birth plan, so they may have to deviate to drugs instead. Certain side effects might be less likely to happen when following the guidelines and with successful planning and counseling, especially concerning women’s individual needs and risks [115]. We have summarized the main side effects of NPPM in Table 4.

**Table 4:** Summary of NPPM techniques’ side effects categorized based on mechanism of action and the supporting references.

Method	Category of Action	Authors Name; Year
**Physical Non-Pharmacological Techniques**		
Massage	▪ Deep tissue massage in sensitive areas like the lower back can cause pain. ▪ Applying massage for too long or too strongly could make laboring women tired and worn out, which inversely impacts the birth experience. ▪ Some oils, lotions, or other things used in massages have been linked to allergic reactions.	Pawale et al. [30]; 2020 Sindle et al. [119]; 2021
Acupressure	If too much pressure is used ▪ Patients may suffer discomfort or pain. ▪ Bruising or soreness at the acupressure point. ▪ Risk of infection if proper hygiene at the acupressure point is not followed.	Tan et al. [120]; 2015 Torkiyan et al. [121]; 2021
Transcutaneous electrical nerve stimulation (TENS)	▪ The electrodes used in TENS may irritate the skin. ▪ Muscle twitching or spasms triggered by stimulation can make the mother uncomfortable. ▪ TENS attachments may interfere with devices assessing fetomaternal wellbeing, making their readings unreliable. ▪ TENS requires active communication by the mother to control the intensity and frequency, which may distract the mother.	Johnson et al. [122]; 2007 Mokhtari et al. [123]; 2020 Njogu et al. [124]; 2021
Water immersion	▪ Infection risk: if there are any tears or open wounds on the mother’s body, water bacteria from the water can enter her bloodstream. ▪ Neonatal aspiration and respiratory distress. ▪ Limits certain medical interventions and devices assessing fetomaternal wellbeing.	Cluett et al. [39]; 2018 Cooper et al. [125]; 2018 Maude et al. [126]; 2020
Heat and cold therapy	▪ Burns, hypothermia ▪ Prolonged exposure to extreme temperatures can be uncomfortable; the mother may discontinue therapy. ▪ The skin may be irritated or become red; itching, or an allergic reaction was reported. ▪ Cold therapy can constrict blood vessels and reduce blood flow to the treated area.	Türkmen et al. [127]; 2021 Didevar et al. [128]; 2022
Breathing techniques	▪ Hyperventilation may cause maternal dizziness, headedness, tingling, and sometimes fainting. ▪ Prolonged breathing exercises stimulate rapid heartbeat, which is distressing to the mother and can cause maternal exhaustion.	Yuksel et al. [51]; 2017 Issac et al. [50]; 2023
**Psychological Non-Pharmacological Technique**		
Relaxation techniques	▪ Limited effectiveness for high-risk pregnancies. So often, they need further techniques. ▪ The inability to achieve the desired relaxation level leads to anxiety or frustration.	Kaple et al. [129]; 2023 Smith et al. [15]; 2018
Virtual Reality (VR)	▪ Creates an immersive environment that may disconnect the mum and cause awareness loss, which is problematic if a response is needed in emergencies or complications. ▪ VR can have the opposite effect on some women, as it may increase pain, anxiety, and fear. ▪ Motion sickness or discomfort due to the sensory disconnect between what they see in virtual and reality.	Kirca et al. [130]; 2023 Carus et al. [131]; 2022 Kılıç et al. [132]; 2023
Music	▪ Create an overwhelming environment and if music is too high, it may increase stress. ▪ Some mothers have a positive attitude to music, while others find it distracting or even offensive owing to different cultural differences.	Santiváñez et al. [133]; 2020 Chehreh et al. [134]; 2023 Surucu et al. [135]; 2018
Distraction	▪ Reduced awareness makes communication with healthcare staff difficult and causes difficulty in: ▪ -Monitoring fetomaternal wellbeing. ▪ -Delay in diagnosis of complications ▪ Distraction techniques may interfere with natural hormone release, especially oxytocin, which exerts a vital role in labor progression and bonding between mother and baby.	Yurtsev et al. [136]; 2021 Amiri et al. [71]; 2019
**Complementary and Alternative Approaches**		
Hypnosis	▪ Ineffectiveness for some women; having unrealistic expectations increases disappointment or frustration. ▪ Some mothers may find the idea of losing control unacceptable, and relying solely on hypnosis may delay critical interventions for any complications. ▪ There is limited research on hypnosis’s potential long-term consequences, especially concerning post-partum depression or anxiety.	Beevi et al. [137]; 2019 Babbar et al. [138]; 2021 Azizmohammadi et al. [139]; 2019
Aromatherapy	▪ Some mothers may be allergic to essential oil or fragrance used, thus causing skin rash or irritation. ▪ Strong aromas may induce nausea, headaches, or even feelings of dizziness. ▪ Certain scents may trigger the opposite effect in mothers due to personal variation. ▪ Aromatherapy can mask significant odors that medical staff rely on for following labor progress.	Kendall et al. [140]; 2018 Tanvisut et al. [86]; 2018
Dancing	▪ Fatigue and risk of injury are increased by jumps and twists. ▪ Distraction from medical interventions. ▪ Unacceptable for some due to cultural considerations.	Akin et al. [141]; 2022 Akin et al. [84]; 2020
Photomodulation	▪ Local reactions include skin irritations, increased temperature, and eye damage. ▪ Some frequencies of the light used in photo modulation devices may interfere with pacemakers or defibrillators.	Traverzim et al. [89]; 2018 Badger et al. [142]; 2017

### 5.5. Further Research and Future Perspective

As we continue to explore the realm of NPPM techniques in labor, it becomes increasingly clear that our understanding is still evolving. The promising results from existing studies highlight the potential of these methods, but they also underscore the need for further rigorous research. This work only included studies in the English language, so it is possible to miss some in non-English languages. This study limitation needs to be addressed in future research.

One area that warrants deeper investigation is the long-term impact of NPPM techniques on maternal–fetal outcomes, maternal post-partum recovery, mental health, breastfeeding success, and mother–infant bonding [143]. Another important research direction is testing integrated pain management models that combine pharmacological and non-pharmacological methods. This approach will offer a more holistic approach to labor pain management, considering women’s unique needs and preferences [98]. With the rise of digital health solutions, their role in NPPM deserves further exploration. There may be opportunities to leverage technology to enhance the effectiveness of NPPM, as in virtual reality and mobile apps, which may facilitate relaxation or deliver personalized pain strategies [144,145].

Lastly, addressing the barriers to implementing NPPM in clinical practice should be emphasized, including improving access to NPPM resources, enhancing healthcare provider training, and changing societal and cultural perceptions about labor pain management [115,146].

## 6. Conclusions

In conclusion, managing labor pain is a complex and multifaceted process that requires a comprehensive approach. NPPM techniques offer a promising alternative or complement to traditional pharmacological methods, potentially enhancing the labor experience, reducing side effects, and improving outcomes for both mothers and babies. These techniques leverage the body’s natural pain management mechanisms and promote relaxation, comfort, decreased anxiety, and increased satisfaction with the birth experience. Moreover, they offer the added benefit of minimal side effects and risks, making them an appealing option for most women.

Many challenges and barriers limit NPPM implementation: limited access, lack of knowledge and education, cultural and societal expectations, and individual preferences. All pose barriers to their usage and require strategies to tackle them. Larger standardized RCTs can improve our understanding of the mechanisms underlying NPPM techniques, assess their efficacy, and explore the long-term complications of using them in childbirth.

In essence, NPPM techniques represent a significant advancement in labor pain management. By embracing these methods and addressing the challenges associated with their implementation, healthcare providers can help to ensure a more positive and empowering labor experience for women.

## Figures and Tables

**Figure 2:**
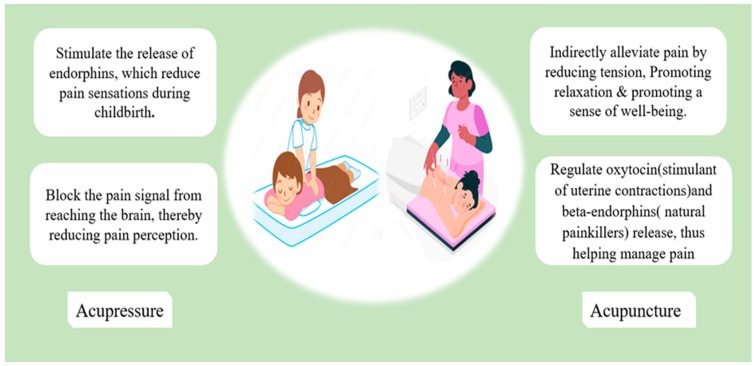
The mechanisms that underlie acupressure’s positive effect in reducing labor pain.

**Figure 3:**
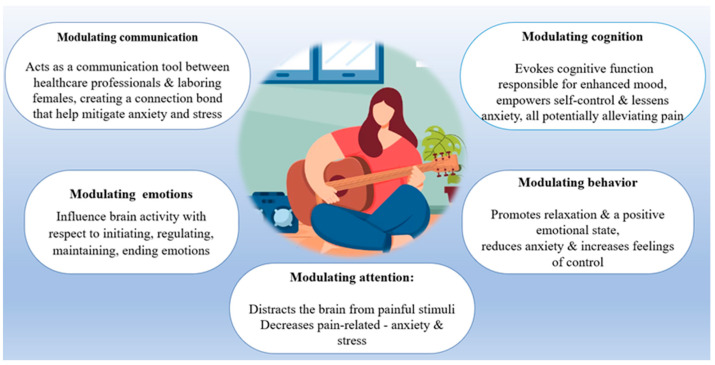
The main pathways by which music conducts its beneficial effect in alleviating labor pain.

**Table 3:** Summary of non-pharmacological pain management in labor: An in-depth analysis of complementary and alternative approaches concerning the mechanism of action, perceived benefit, and the supporting references.

**Methods**	**Methods Sub-Types**	**Proposed Mechanism of Action**	**Perceived Benefit**	**Authors’ Name; Publication Year**
Hypnosis	▪ Natural hypnosis ▪ Self-hypnosis ▪ Stage hypnosis ▪ Hypnotherapy	▪ Modulates pain intensity caused in the primary somatosensory cortex. ▪ Relaxes and distracts attention from the pain sensation.	▪ Reduces net use of analgesia during childbirth. ▪ No clear evidence of pain satisfaction relief or coping sensation	Madden et al. [77]; 2016 Cyna et al. [78]; 2013 Downe et al. [79]; 2015
Integration of religion/health and well-being	▪ Praying, ▪ Reciting the Quran, ▪ Fasting, ▪ Islamic meditation (dhikr) has been shown to relieve stress, improve health, increase productivity, and enhance quality of life.	▪ Distracts and inhibits the pain perception.	▪ It efficiently reduced labor pain and improved the score of pain behaviors.	McLaren H et al. [80]; 2021 Desmawati et al. [81]; 2019 Kocak et al. [82]; 2022
Dancing	--	▪ Dancing combines the beneficial effects of music and the effects of upright position and movements, such as pelvic tilting and rocking.	▪ Mean scores of pain were lower. ▪ The level of birth satisfaction was significantly higher.	Abdolahian et al. [83]; 2014 Akin et al. [84]; 2020
Aromatherapy	Essential oils may be given as: ▪ Massage into the skin, ▪ In a warm bath, ▪ Diffused into the air using a diffuser	▪ Limbic system stimulation; releases serotonin and endorphins. Thereby reduces anxiety and tension, leading to lower pain perception. ▪ Augmenting the production of endogenous stress-alleviating substances within the human body. Such as decreasing cortisol and/or increasing serotonin. ▪ Some essential oils possess direct pain-relieving effects, such as rosemary.	▪ Trend decrease in labor pain. ▪ Trend decrease in anxiety during labor	Tabatabaeichehr et al. [85]; 2020 Sirkeci et al. [86]; 2023 Hamdamian et al. [87]; 2018
Photomodulation	--	Irradiation induces. ▪ Neural block and modulates neurotransmitters. ▪ Reduced muscle spasms. ▪ Reduced interstitial edema, thereby exhibiting anti-inflammatory effects	▪ Pain reduction ▪ Analgesic effect	Traverzim et al. [88]: 2021 Traverzim et al. [89]; 2018
Support therapy	▪ Emotional Support ▪ Advocacy ▪ Informational Support	▪ Reduces stress and anxiety by providing reassurance and empathy, thus decreasing pain perception. ▪ Supports women’s autonomy and decision-making, understanding of the labor process, promoting feelings of control and confidence	▪ Reduced perception of pain via reduced stress and anxiety ▪ Decreased use of analgesics, ▪ ncreased overall satisfaction with the birthing process.	Akbas et al. [90]; 2022 Bohren et al. [91]; 2017 Ip et al. [92]; 2009

## Data Availability

Not applicable.
